# Access Control Development Within the Framework of an IOTA-Based Electronic Medical Record Management System

**DOI:** 10.3390/s26051422

**Published:** 2026-02-24

**Authors:** Hari Purnama, I Putu Bakta Hari Sudewa, Tazkia Nizami, Bagas Sambega Rosyada, Pradipta Rafa Mahesa, Nur Ahmadi

**Affiliations:** School of Electrical Engineering and Informatics, Bandung Institute of Technology, Bandung 40132, Indonesia; baktaharisudewa21@gmail.com (I.P.B.H.S.); tazkia.nizami@gmail.com (T.N.); bagassambega@gmail.com (B.S.R.); 13522162@std.stei.itb.ac.id (P.R.M.); nahmadi@itb.ac.id (N.A.)

**Keywords:** internet of things, access control, IOTA, hierarchical identity-based encryption (HIBE), hierarchical access control

## Abstract

Electronic Medical Records (EMRs) are mandatory in Indonesia following the Ministry of Health regulation, which raises significant challenges in data security and patient-centric access control. Current implementations rely on centralized healthcare systems or third-party vendors, creating risks of unauthorized access, data leakage, and uncertain data integrity. To address these issues, this study proposes DecMed, a decentralized EMR management framework built on IOTA Distributed Ledger Technology (DLT). DecMed integrates Capability-Based Access Control (CapBAC), Proxy Re-Encryption (PRE), and the InterPlanetary File System (IPFS) to enforce patient ownership of medical data. Patients actively grant or revoke access, define access duration, and selectively share data with healthcare personnel. The system is implemented using smart contracts in the Move programming language on the IOTA ledger, while encrypted clinical data is stored on IPFS. Evaluation through unit testing of various unauthorized access scenarios demonstrates that DecMed effectively enforces fine-grained access rules, preserves data confidentiality and integrity, and ensures compliance with national healthcare requirements.

## 1. Introduction

In Indonesia, medical records are an important part of healthcare services because they provide long-term information on patients’ conditions, diagnoses, and treatments. The Republic of Indonesia Health Minister Regulation No. 24 of 2022 requires all healthcare facilities to maintain medical records in electronic form. The regulation also requires that confidentiality, integrity, and availability be maintained. This comprehensive longitudinal information can be used to improve the community’s quality of life by revealing patterns in symptoms, disease progression, and how this data can support more effective and efficient health interventions [1].

The healthcare data landscape in Indonesia is currently fragmented. Each healthcare facility operates its EMS (Electronic Medical Record) system independently, resulting in patient data scattered across institutions. This fragmentation not only disrupts continuity of care but also limits analysis, potentially complicating and even reducing the accuracy of clinical decision-making at scale across healthcare facilities. Fundamentally, this fragmentation reflects a governance challenge: healthcare institutions operate at relatively equal organizational levels, and there is no single entity that can naturally be designated as the authoritative controller of medical data across the ecosystem [2].

Although various interoperability initiatives have been developed to enable data exchange between heterogeneous systems, these approaches have not fully addressed fundamental governance issues. Centralizing control in a single entity, such as a government agency, can create a single point of failure, leading to risks of imbalance in trust, limited scalability, and long-term system sustainability on a national scale. In this context, fundamental questions arise about which party has the authority to determine access decisions, enforce accountability, and maintain trust among independent organizations that share data management responsibilities.

The Indonesian government has introduced the SATUSEHAT platform as a national health data ecosystem to address these interoperability issues. The platform functions as an application that facilitates the exchange of standardized data between health information systems [2]. However, SATUSEHAT does not implement a uniform access control mechanism across all participating institutions. The platform also operates at the level of data aggregation and interoperability. As a result, access control policies are typically implemented independently by each healthcare facility or third-party vendor, resulting in heterogeneous, institution-centric authorization [3]. These mechanisms lead to inconsistencies in how patient consent is applied, how access is audited, and how trust is built across organizations.

Access control emerges as a key governance challenge in cross-institutional EMR environments. Regulations state that patients retain ownership rights over their medical data and that access must be granted only with explicit consent [1]. In practice, EMR platforms and health data often adopt a coarse and relatively static authorization approach (e.g., role-based access with tiered access levels), which is inadequate for the fine-grained, context-aware, and delegable access required for real-world healthcare scenarios [4,5,6,7]. These limitations are influential in multi-institutional care environments, emergency access scenarios, and dynamic delegation among medical personnel.

Management of EMR systems also requires reliable and verifiable audit trails. Audit logging is a core component of audit controls, and is generally required to support compliance and incident investigations [8,9]. Audit records are also an important source of data for detecting and investigating abuse by internal parties [10]. Emergency access (“break-glass”) mechanisms must be accompanied by accountability and compliance with policies. Therefore, the need for auditable access trails is becoming increasingly crucial [11].

The consent aspect adds complexity to access management. The current consent mechanism is synchronous, requiring direct interaction with patients when accessing data. This design is impractical when access must be delegated among various parties in the healthcare system or when patients are unable to give consent due to emergency conditions. Previous research on user-managed consent models emphasizes the importance of separating policy definition, authorization decision-making, and enforcement while maintaining transparency and patient control [12,13]. However, this approach assumes a centralized trust infrastructure, limiting its applicability in decentralized, cross-institutional healthcare environments.

In this framework, Decentralized Ledger Technology (DLT) has been studied and applied to electronic medical record systems to address issues of access governance, trust fragmentation, and auditability. Several studies show that DLT-based EHR and PHR architectures integrate access control, approval policies, and audit mechanisms directly into the ledger layer via smart contracts or protocol-level rules. This approach differs from conventional models that place access control as a component dependent on specific institutions. The use of DLT enables the implementation of verifiable access mechanisms, supports transparent delegation processes, and provides tamper-proof recording of medical data access across organizational boundaries [14,15,16].

A systematic review shows that blockchain-based health record systems are highly effective in environments with autonomous healthcare providers. In this context, DLT facilitates trust between institutions through algorithmic consensus and joint verification of access events. At the same time, sensitive medical data remains stored outside the blockchain to maintain privacy and scalability [17,18]. This empirical implementation highlights the role of DLT as an infrastructure that facilitates governance, transforming the way access control, accountability, and trust operate within distributed healthcare ecosystems.

Based on these observations, this study argues that integrating a patient-oriented access control framework with a DLT-based governance layer is a promising approach to overcoming institutional fragmentation in EMR systems. The proposed approach does not only focus on data storage or interoperability, but rather emphasizes strengthening access governance, auditability, and patient sovereignty across healthcare institutions. In this context, DLT is used to shape access controls, while sensitive medical data is stored off-chain to maintain privacy and scalability.

Although interest in blockchain-based health record systems continues to grow, understanding of how distributed governance infrastructure options fundamentally change access control design in cross-institutional healthcare environments remains limited.

Accordingly, this study is guided by the following research questions:

- RQ1: How can a patient-centric access control framework be designed to enable cross-facility medical record integration while preserving data sovereignty and auditability?
- RQ2: What role can Distributed Ledger Technology play in shaping, enforcing, and verifying access governance across independent healthcare institutions?

To address these questions, this paper proposes DecMed, a distributed EMR management framework that integrates access control mechanisms with a DLT-based governance layer. The framework focuses on defining architectural principles for granular, revocable, and delegable access control, supported by tamper-evident audit trails. While the proposed system leverages IOTA as the underlying distributed ledger due to its suitability for high-throughput environments, the primary contribution of this work lies in the design of an access control framework for distributed medical record management rather than in the specifics of a single platform.

The contributions of this study can be summarised as follows:

- A governance-oriented analysis of institutional fragmentation in cross-institution EMR systems, highlighting access control and auditability as core challenges.
- A patient-centric access control framework that supports granular consent, delegation, and revocation across healthcare facilities.
- An architectural approach that integrates a DLT-based governance layer (implemented using IOTA) to enable verifiable access governance and tamper-evident audit trails without centralized data ownership.

## 2. Background

In this section, we provide a concise introduction to key concepts: electronic medical record, proxy re-encryption, capability-based access control, and IOTA.

### 2.1. Electronic Medical Record (EMR)

According to Article 1, paragraphs (1) and (2) of the Regulation of the Minister of Health of the Republic of Indonesia No. 24 of 2022 on Medical Records [1], “A Medical Record is a document containing patient identity data, examination, treatment, procedures, and other services that have been provided to the patient,” while “An EMR is a Medical Record created using an electronic system intended for the implementation of Medical Records.” Based on this definition, medical records are a fundamental aspect of the healthcare process in Indonesia. Moreover, the concept of a medical record is not merely a form of medical information storage for a patient, but also represents an ongoing process from the moment the patient registers until treatment is completed, as well as how the record is accessed, stored, and distributed to certain parties for specific purposes [19].

Under Law No. 19 of 2016 on Electronic Information and Transactions, EMRs are classified as electronic documents. EMR data must be created and managed within an electronic system that ensures security and accountability, so that it can serve as legal evidence [20]. In the implementation of medical records, whether manual or electronic, legal aspects must be carefully considered. This is crucial to ensure that all parties involved in hospital medical or healthcare services receive adequate legal certainty and protection. EMR is a mandatory component that must be implemented by all healthcare facilities (fasyankes) in Indonesia. These facilities include [1]:

- Independent practices of doctors, dentists, and/or other health professionals;
- Community Health Centres (Puskesmas),
- Clinics,
- Hospitals,
- Pharmacies,
- Health laboratories,
- Public health centres (Balai), and 
- Other healthcare facilities designated by the Minister of Health of the Republic of Indonesia.

EMR is also considered a subsystem that is eventually integrated with other subsystems within a healthcare facility [1].

The contents of the EMR, according to Article 27, paragraph (1) of the Regulation of the Minister of Health No. 24 of 2022, consist of:

- Administrative documentation, and 
- Clinical documentation

According to Article 27, paragraph (2) of the same regulation, administrative documentation must, at a minimum, include registration documentation. Based on Article 14, paragraph (1), registration documentation consists of the identity and social data of patients receiving outpatient, emergency, and inpatient care. According to Article 14, paragraph (2), identity data must at a minimum include the medical record number, patient name, and National Identification Number (NIK). According to Article 14, paragraph (4), social data must at minimum include religion, occupation, education, and marital status [1].

The Decree of the Minister of Health No. HK.01.07/MENKES/1423/2022 provides a more detailed breakdown of the EMR variables and metadata, which are grouped into five categories: emergency care, outpatient care, inpatient care, laboratory, and pharmacy [21].

### 2.2. Capability-Based Access Control (CapBAC)

The main concept of CapBAC is the notion of a capability. A capability is an object that contains access rights. This object can then be granted to an entity, allowing that entity to access data owned by another entity [22]. Each data-owning entity creates capabilities and can either distribute them directly to the intended recipient or store them in a location that guarantees their integrity.

CapBAC offers fine-grained access control, as each capability can include various access attributes such as the metadata of the access provider, the portion of data that can be accessed, the type of access, and even the expiration time of the access. Moreover, CapBAC is well-suited to decentralized environments, as it does not require a centralized authority to validate or grant access. DLT, with its immutability, is one of the most suitable technologies to integrate with CapBAC, as it can serve as a storage medium for capabilities.

### 2.3. Access Control Comparison

Table 1 shows a Comparison between other—Role-Based Access Control (RBAC) and Attribute-Based Access Control (ABAC)—with the CapBAC method based on various requirements as defined by Ahsan et al. [23].

- Granularity. Refers to the ability to manage access to data and resources by creating customized access control policies for each user or group. These policies typically require contextual details to enable precise control and should ideally be articulated more explicitly. Specifically for CapBAC, granularity can be enhanced by adding additional attributes within the created capability [22].
- Interoperability. Refers to the ability to effectively coordinate access control mechanisms across heterogeneous domains, which may involve a variety of devices, protocols, networks, and platforms.
- Delegation. Refers to a scenario in which an entity with authority over a resource or device can grant access rights (called the delegator) or temporarily delegate certain permissions to another entity (called the delegatee) under predefined constraints.
- Automatic revocation. Refers to the act of dynamically removing or cancelling access rights from a user or device.
- Scalability. Scalability is the ability of a network to expand. To effectively handle the heterogeneity and evolving nature of devices, resources, and services, an access control model must generally be highly adaptable in both the scope and format of its policies.
- Distributed nature. Refers to the need for the system to operate effectively in environments without a single central control point.
- Ease of use. Refers to how easy it is to use the access control system, for example, in terms of implementation simplicity and access logic.

### 2.4. Proxy Re-Encryption (PRE)

PRE leverages a semi-trusted proxy to transform a ciphertext into a new ciphertext that retains the same content but can be decrypted only with the secret key of a designated subject. The concept of PRE was first introduced by Blaze et al. in 1998. As synthesized from several sources by Qin et al., the general intuition behind the PRE mechanism can be described as follows [24,25].

The initial ciphertext is produced by encrypting the data using the delegator’s public key. This ciphertext can then be stored in a storage location, such as cloud storage or a DLT system. When a delegatee requests access to the encrypted data, the delegator generates a re-encryption key using their own secret key and the delegatee’s public key. This re-encryption key is sent to the proxy, which uses it, together with the original ciphertext and the public keys of both parties, to perform the re-encryption. The result is a new ciphertext that can be decrypted only with the delegatee’s secret key.

Even though PRE limits the proxy’s ability to learn the plaintext, the architecture still centralizes re-encryption functionality in a single semi-trusted entity, effectively creating a single point of failure: if the proxy malfunctions or becomes the target of a denial-of-service (DoS/DDoS) attack, the re-encryption process is halted. The entire authorization or key distribution workflow becomes unavailable to all dependent subjects. This weakness of single-proxy PRE has been recognized in the literature as a fundamental limitation [26].

### 2.5. InterPlanetary File System (IPFS)

IPFS is a peer-to-peer (P2P) distributed file system that aims to connect all computing devices to a single file system [27]. Unlike the traditional Hypertext Transfer Protocol (HTTP), which uses location-based addressing (identifying data by where they are stored, e.g., IP address or domain name), IPFS utilizes content-based addressing. This means that data are identified and retrieved based on their cryptographic hash, known as the Content Identifier (CID).

When a file is added to the IPFS network, it is split into smaller chunks, cryptographically hashed, and given a unique CID. This CID acts as a permanent, immutable fingerprint of the data. If the file’s content changes, the cryptographic hash changes, resulting in a completely new CID. This immutability feature makes IPFS highly compatible with DLTs, ensuring data integrity [27].

In the context of healthcare data storage, IPFS enables off-chain storage of large, encrypted medical files, while only small CIDs need to be stored on the immutable ledger (on-chain). This approach overcomes the storage size limitations typically found in DLT or blockchain networks while maintaining a decentralized architecture without a single point of failure.

### 2.6. IOTA

IOTA is a type of DLT that adopts a Directed Acyclic Graph (DAG)-based network. DLT functions as a decentralized system that allows data to be recorded and stored across multiple nodes in a network, ensuring that all ledger copies remain synchronized and consistent with one another [28]. DLT eliminates the need for a centralized authority, thereby enhancing transparency within the system [29]. This technology is widely used across various sectors, including finance, supply chains [30], healthcare [16], and energy [29].

The DAG network structure enables IOTA to support high throughput, as it can validate new transactions more quickly than conventional blockchains such as Bitcoin or Ethereum [31,32]. Initially (IOTA 2.0), IOTA did not impose transaction fees (zero-cost transactions). This feature made IOTA ideal for microtransactions and usage in lightweight environments such as Internet of Things (IoT) devices, where efficiency and speed are critical factors [31,32]. However, in IOTA Rebased [33], the zero-cost transaction model no longer applies. Each transaction now incurs a gas fee [34].

### 2.7. IOTA Rebased

IOTA Rebased marks a significant evolution of the IOTA protocol, transitioning from a Tangle-based structure that supported zero-cost transactions and relied on a centralized coordinator, to an object-based ledger built on a DAG. This new approach is designed to enhance scalability and programming flexibility, particularly for IoT applications and enterprise needs [33,35]. Unlike IOTA 2.0, which was based on the Unspent Transaction Output (UTXO) model, IOTA Rebased adopts an object-based DAG inspired by the Sui Network architecture. This structure enables parallel transaction processing and supports complex transaction patterns through fine-grained access control [35].

The ledger in IOTA Rebased distinguishes between shared objects, which can be accessed and modified by multiple entities based on predefined rules, and owned objects, which are fully controlled by a single entity. This concept paves the way for advanced programmability, such as in supply chain tokenization scenarios [35]. This architectural shift aims to overcome various limitations present in IOTA 2.0, particularly delayed decentralization and the lack of smart contract support on Layer 1 (L1) [33].

Consensus in IOTA Rebased is powered by the Mysticeti protocol, a Byzantine Fault Tolerant (BFT) system implementing Delegated Proof-of-Stake (DPoS) to achieve high throughput and low latency [33,35]. Mysticeti provides finality in approximately 500 ms and supports more than 50,000 TPS. This is made possible by using an uncertified DAG, which eliminates the need for explicit certificates and completes block commitment in just three message rounds [35]. Validators are selected via staking, with an initial 150 committee slots. These validators are responsible for transaction validity and finality, while the protocol dynamically identifies malicious blocks through pattern analysis on the DAG to maintain network integrity [35].

### 2.8. IOTA Gas Station

The IOTA Gas Station simplifies user interaction with the IOTA network through sponsored transactions [36]. Sponsored transactions eliminate the need for users to manage tokens for paying transaction fees. The architecture of the IOTA Gas Station consists of three components: the Gas Station Server, Gas Station Storage, and the Key Store Manager (KSM). The Gas Station Server is the core component of the Gas Station system. It provides two main API endpoints—one for reserving gas objects and another for executing transactions using previously reserved gas objects [36].

Multiple Gas Station Servers can be deployed together to form a Gas Station Pool. Servers in the pool can share the sponsor address and persistent storage. The Gas Station uses the sponsor address to store IOTA tokens. These tokens are then divided into smaller tokens (gas objects) and used to sponsor transactions [36].

Gas Station Storage serves as the persistent storage for saving gas objects and the state of sponsored transactions. Redis is used as the underlying database. Additionally, LUA scripts are implemented to enable concurrent access from multiple Gas Station Servers, thus improving system performance and scalability [36].

The KSM (Key Store Manager) manages keys for signing transactions. There are two KSM configurations: External Key Management Service (KMS) and in-memory key storage. KMS uses external services such as AWS KMS to enhance security. In-memory key storage, as the name suggests, stores keys directly in the Gas Station Server instance. While this is less secure, it offers better performance. Overall, the IOTA Gas Station enables easier access to the IOTA network, particularly for systems that do not implement their own token mechanism. Users do not need to manage tokens to perform transactions on the IOTA network [36].

## 3. Related Work

Prior research on Electronic Medical Record (EMR) and Personal Health Record (PHR) systems has extensively explored challenges related to privacy, access control, interoperability, and trust management. Existing works can be broadly categorized into three main research themes: (1) privacy-aware and consent-driven EMR frameworks, (2) blockchain and Distributed Ledger Technology (DLT)-based health record systems, and (3) access governance and auditability in decentralized healthcare environments.

### 3.1. Privacy-Aware and Consent-Driven Health Record Frameworks

A significant body of research emphasizes the importance of embedding privacy and consent management into the design of health record systems. Semantha et al. [3] propose a conceptual framework that integrates privacy-by-design principles with regulatory requirements such as GDPR and national privacy guidelines. Their work highlights common privacy risks in patient record management, including data breaches and unauthorized secondary use of medical data, and argues that patients should retain meaningful control over access to their records through explicit consent mechanisms.

While such frameworks provide valuable conceptual guidance for privacy-aware EMR design, they typically assume centralized system architectures and rely on institution-managed databases. As a result, issues related to single points of control, limited transparency, and cross-institution trust remain unresolved. Moreover, these approaches often lack concrete implementation and evaluation in distributed healthcare settings, limiting their applicability in multi-institutional environments.

### 3.2. Blockchain and DLT-Based Health Record Systems

To address the limitations of centralized architectures, a growing number of studies explore the application of blockchain and DLT in health record management. Several works demonstrate how distributed ledgers can enhance data integrity, traceability of access, and trust among independent healthcare providers. Shahnaz et al. and Yaqub et al. show that blockchain-based architectures can support secure sharing of medical records by recording access events and permissions in an immutable ledger [14,15].

Akbulut et al. further extend this line of research by proposing an IOTA-based PHR access management system that integrates distributed ledger technology, decentralized storage, and proxy re-encryption to support fine-grained access delegation. Their prototype demonstrates that DLT can serve as more than a passive data store, instead actively enforcing access policies and recording audit trails across multiple actors [16].

Systematic reviews and surveys reinforce these findings. Han et al. and Basu et al. analyze a wide range of blockchain-based EHR systems and conclude that DLT is particularly well-suited for healthcare ecosystems involving multiple autonomous organizations without a universally trusted central authority. These studies consistently emphasize the role of blockchain in enabling tamper-evident logging, distributed trust establishment, and policy enforcement, while keeping sensitive medical data off-chain for privacy and scalability [17,18].

### 3.3. Access Governance and Auditability in Decentralized Healthcare Systems

Beyond data sharing and data integrity, recent research increasingly treats access governance, auditability, and accountability as first-class concerns in distributed healthcare systems. Prior studies observe that traditional role-based and institution-centric access control models are difficult to extend toward dynamic delegation, emergency access, and cross-facility accountability in multi-organizational healthcare environments [14,15].

Blockchain- and DLT-based approaches address these limitations by embedding access control logic, consent policies, and audit mechanisms directly into the ledger layer through smart contracts or protocol-level rules, rather than relying on institution-specific enforcement [16]. In such systems, access decisions and policy enforcement become verifiable and independently auditable by participating institutions, thereby reducing reliance on centralized authorities and increasing cross-organizational trust [17].

This governance-oriented use of DLT aligns with the concept of *law-by-code*, in which system behavior and compliance requirements are enforced algorithmically rather than solely through organizational trust. Systematic reviews further indicate that these properties are particularly relevant for national-scale healthcare ecosystems characterized by institutional plurality and the absence of a universally trusted central authority [18].

### 3.4. Research Gap

Although prior studies demonstrate the potential of DLT for enhancing privacy, integrity, and access traceability in health record systems, several gaps remain. Existing works often focus either on conceptual privacy frameworks without distributed enforcement, or on technical DLT implementations without sufficient alignment to national healthcare regulations and governance requirements. In particular, mechanisms for patient-centric access control, explicitly designed to operate across independent healthcare facilities while remaining compliant with local regulatory frameworks, such as those in Indonesia, remain underexplored.

This study addresses these gaps by proposing DecMed, a distributed EMR management framework that integrates patient-centric access control with a DLT-based governance layer. Unlike prior works that treat access control as an auxiliary feature, DecMed places access governance, auditability, and patient sovereignty at the core of the system design, while remaining adaptable to national regulatory constraints.

## 4. Proposed Design

This section presents the proposed design that leverages IOTA to enhance EMR management.

### 4.1. Scope & Planning

Prior to beginning the design phase, it is crucial to define the scope and develop a clear plan. This step guarantees that the design stays focused on the research goals and avoids incorporating irrelevant aspects. Moreover, careful planning reduces the risk of missing important elements after the design is completed.

Scope.First, the system is designed exclusively in accordance with the regulations, standards, and existing systems applicable in Indonesia. This focus ensures that the proposed framework complies with national legal and healthcare requirements, making it directly relevant and implementable within the Indonesian healthcare context.

Second, this research does not address unauthorized access resulting from users losing their credentials. Handling such cases, including credential recovery and related security protocols, is beyond the scope of this study and would require a separate, dedicated investigation.

Choosing access control. In an EMR management system, access control is a critical aspect to ensure the security of EMR data. The access control mechanism must cover the various access scenarios performed by actors involved in healthcare services. These actors include patients as owners of EMR data, healthcare facility personnel, and the Ministry of Health, the healthcare regulator in Indonesia. Patients must give active consent before other parties can access their EMR data during medical examinations.

The granting of consent or delegation of access must also consider the duration of access and the specific parts of the EMR data that can be accessed. Administrative personnel, who are generally responsible for managing EMR data within a healthcare facility, may only view the administrative portion of a patient’s EMR. Medical personnel, such as doctors and dentists, are permitted to access both the administrative and clinical portions of the patient’s EMR data to perform diagnoses, determine therapy, and make other medical decisions. All access actions are limited to a certain duration based on the healthcare service needs. Furthermore, the access control mechanism must support scenarios in which patients can revoke access easily and effectively.

There are six key requirements to consider when selecting an access control mechanism. These requirements include:

- Granularity of control.High granularity allows for more specific access rights (e.g., only certain parts of the data), ensuring alignment with user needs while maintaining data security.
- Support for distributed environments. Support for distributed environments is crucial to facilitate seamless integration of access control with DLT.
- Ease of access revocation. The ability to easily revoke access is essential for meeting system needs, particularly when a data owner wishes to withdraw previously granted access.
- Ease of access delegation. Access delegation is a core mechanism of the developed system. A data owner must be able to easily delegate access to their data, either directly to another party or to a third party.
- Ease of implementation and access validation logic. Simple access validation logic reduces the risk of implementation errors. Furthermore, straightforward validation logic can also improve system performance.

Based on these requirements and the comparison presented in Section 2.3, *CapBAC* was selected as the basis for the DecMed system’s access control mechanism.

Choosing DLT. Based on the background presented in Section 1, DecMed provides an access control solution that handles these scenarios by leveraging DLT. The chosen DLT must support a sufficiently high TPS rate and low time to finality to handle all requests promptly. Additionally, managing transaction gas fees is an important consideration because, in a non-financially focused system like DecMed, actors involved should not be required to hold tokens merely to perform transactions.

Two DLT alternatives were considered: Hyperledger Fabric and IOTA. These options were selected based on their relevant features and successful application in previous research efforts addressing similar challenges [36,37]. A comparative analysis of both technologies was conducted using the criteria previously explained, and the results are presented in Table 2. Based on this comparison, IOTA was chosen as the most suitable DLT for the system. IOTA offers high throughput and fast time to finality, enabling it to handle a large number of requests efficiently. Furthermore, the IOTA Gas Station, which serves as an official tolling mechanism for sponsored transactions, simplifies gas fee management—particularly important for systems like DecMed that do not rely on tokens for transaction execution.

Partitioning data for efficiency and security. Besides access control, the integrity of the EMR data stored is another crucial aspect. The immutable nature of DLT significantly enhances data integrity. However, DLT has limitations on the amount of data that can be stored in a single transaction, and storing large amounts of data on the DLT network increases data transfer load. Therefore, it is necessary to carefully partition EMR data into on-chain (within the DLT) and off-chain components.

In addition to immutability, DLT is characterized by transparency, meaning that anyone can read data stored as transactions on the DLT network. As mentioned earlier, EMR data is sensitive and must be accessed by authorized parties with patient consent. When EMR data is stored on the DLT, additional mechanisms are needed to protect it from unauthorized access. This protection involves encrypting EMR data before it is stored on the DLT. Encryption must be applied not only to the on-chain data but also to off-chain data.

Two alternatives were considered for storing EMR data: directly on IOTA nodes or using IPFS. In this system, both options are combined to optimize performance and scalability. IPFS is used to store the encrypted clinical portion of the EMR, while the administrative data and metadata—including CIDs—are stored directly on IOTA nodes. This approach was chosen because the clinical portion can be relatively large, exceeding IOTA’s object size limit of 250 KB. Additionally, IPFS offers a decentralized, scalable storage solution that can be efficiently managed using IPFS clusters.

As previously explained, the EMR data is distributed across IOTA nodes and IPFS. In addition, Redis is used to store auxiliary data required for the PRE mechanism, such as nonces and other cryptographic metadata. The data stored on IOTA includes:

- The administrative portion of patient EMR data,
- Administrative data of healthcare facility personnel,
- Clinical metadata, including encryption keys and IPFS CIDs.

All data stored on IOTA is encrypted before being committed to the network. Meanwhile, IPFS is specifically used to store the encrypted clinical portion of the patient’s EMR.

Defining system actors and privileges. Based on the previous analysis, there are five main actors in the DecMed system. The privileges and access rights of these five actors are outlined in Table 3. Each actor plays a distinct role in the system, and their permissions are defined by their responsibilities and the medical data they require access to. This role-based privilege mapping ensures that sensitive information is accessible only to authorized parties, thereby strengthening security within the DecMed ecosystem.

### 4.2. Design

**Figure 1:**
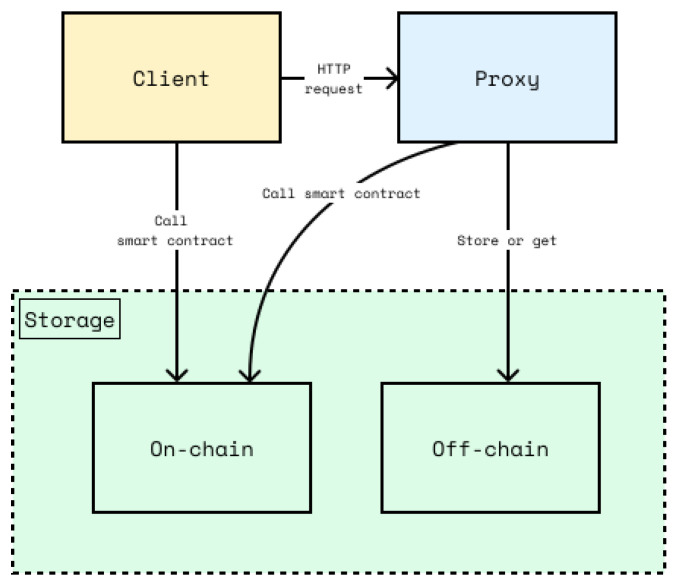
Illustration of the DecMed system description.

The system description is illustrated in Figure 1. The directional arrows in this diagram represent the initiation of communication and component dependencies rather than a strict chronological sequence. For instance, an arrow from the Client to the Proxy indicates that the Client initiates requests, and the Proxy responds; the Proxy does not independently initiate connections to the Client. Detailed sequential workflows are provided in subsequent process diagrams.

The proposed design consists of three main components: the Client, Proxy, and storage. The Client serves as the primary interface for actors to interact with the system, with functionality tailored to each actor’s specific role. The Proxy plays a central role in the EMR data access control mechanism, particularly in the PRE process. All forms of access to a patient’s EMR by other parties must pass through the Proxy.

Communication between the Client and Proxy is established using standard web protocols. While Figure 1 identifies this link as HTTP to denote the application-layer protocol, the actual system implementation should be strictly enforced HTTPS (transporting HTTP over TLS/SSL) to ensure channel security and data confidentiality.

The storage component is divided into two categories: on-chain and off-chain storage. On-chain storage refers to data stored on the DLT network, while off-chain storage refers to data stored outside the DLT. Given its immutability, on-chain storage is crucial for maintaining the integrity of stored EMR data. Meanwhile, off-chain storage is used to store large amounts of data or data with an expiration period that is not immutable.

The Client communicates with the Proxy for purposes such as access delegation, retrieving patient EMR data, or accessing data stored in off-chain storage. Additionally, the Client can interact directly with on-chain storage to access the actor’s private data. The Proxy has direct communication capabilities with both on-chain and off-chain storage. When a request to access a patient’s EMR is made, the Proxy retrieves the relevant data from on-chain storage. This data is then used in the PRE process, enabling the authorized party to access the patient’s EMR.

As previously described, on-chain storage plays a key role in safeguarding EMR data integrity. Every access granted by the patient is recorded as a capability entry in the on-chain storage. The immutability of on-chain storage ensures that unauthorized parties cannot alter these entries. When the Proxy queries the on-chain storage to obtain the data required for PRE, the system verifies the existence of the corresponding access entry through a smart contract. This mechanism forms the foundation of the CapBAC model implemented in the DecMed system.

**Figure 2:**
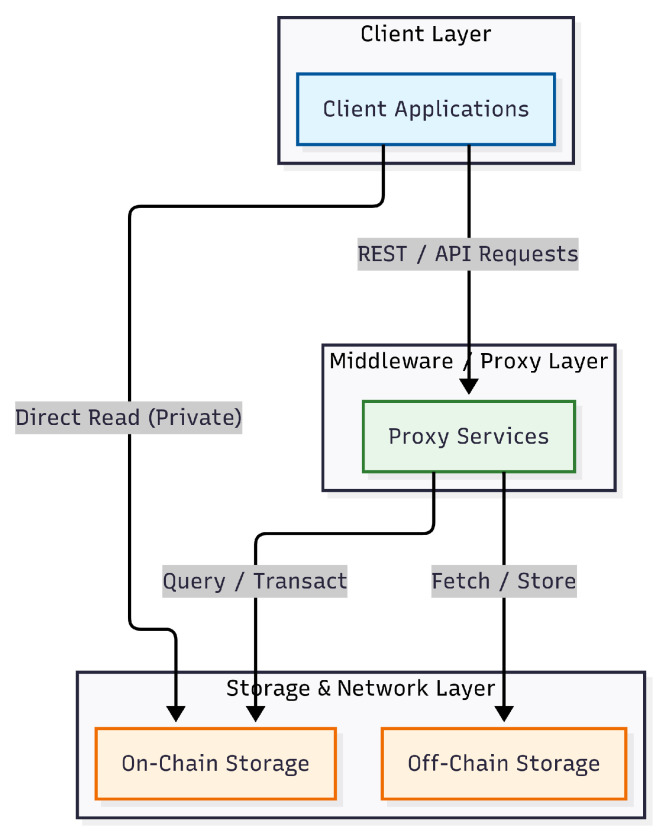
DecMed General System Architecture (Level 0).

The high-level system overview is illustrated in Figure 2, where each arrow shows the direction of dependency. The design adheres to a three-tier architecture comprising the Client Layer, the Proxy Layer, and the Storage Layer.

The Client Layer serves as the primary interface for all actors, routing user inputs to the appropriate backend services. The Proxy Layer acts as the security middleware, enforcing access control policies and managing cryptographic operations such as Proxy Re-Encryption (PRE). Finally, the Storage Layer is bifurcated into on-chain and off-chain components. On-chain storage (DLT) ensures the immutability of access capabilities, while off-chain storage (IPFS) handles high-volume encrypted clinical data.

The specific configuration of the Client Layer and the actor hierarchy is detailed in Table 4. The system defines three distinct client applications tailored to specific platform requirements, ensuring that each user role is strictly mapped to an optimized interface.

**Figure 3:**
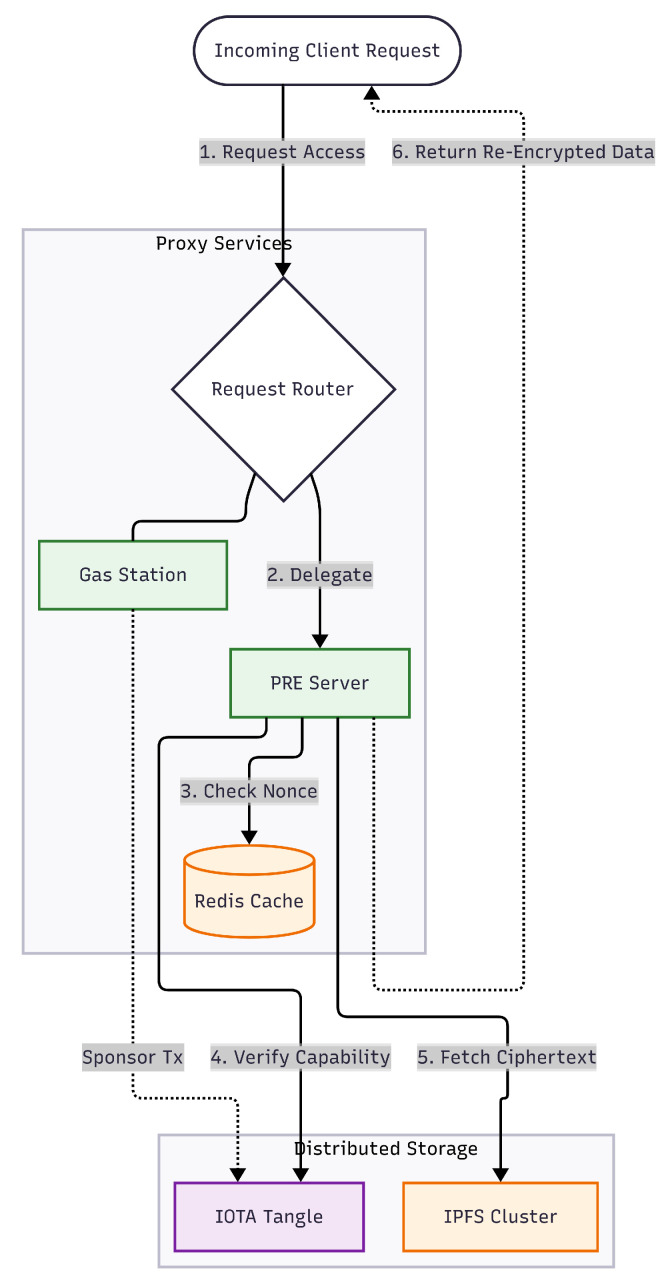
Middleware and Storage Infrastructure (Level 1).

While Table 4 outlines the user-facing client applications, the underlying Proxy and Storage Layers manage the core security, access control, and data persistence logic. The architectural interaction between these backend subsystems is detailed in Figure 3 and consists of the following components:

- PRE Servers: Handle re-encryption. When a data request is received, the PRE server verifies the capability entry on the IOTA network before re-encrypting the data fetched from IPFS for the requester. Redis is utilized here to store ephemeral data, such as nonces, to prevent replay attacks.
- Gas Station Servers: To abstract the complexity of cryptocurrency from the end-user, these servers sponsor transactions on the IOTA network, allowing non-financial actors to interact with the ledger without holding tokens.
- Storage Division: The IOTA Tangle serves as the immutable trust anchor for access control policies (CapBAC), while the IPFS Cluster provides decentralized, content-addressable storage for the encrypted EMR files.

To ensure secure and non-custodial identity management, the Client implements the BIP39 standard [39]. Upon registration, a 12-word mnemonic phrase is generated strictly for account recovery and account sign-in purposes. For daily operations and emergency access, the system uses a local authentication mechanism in which the private key is encrypted and stored in the device’s secure hardware. This allows patients to authenticate using only a PIN or biometrics (fingerprint/face recognition), balancing high-entropy security with the need for rapid access in critical situations.

### 4.3. Standards and Data Model Considerations

This study focuses on access control and governance mechanisms in cross-institution EMR systems rather than on comprehensive modelling of medical record data. Consequently, the structured medical data considered in this work is limited to elements necessary to support authorization decisions, consent management, and auditability.

HL7 Fast Healthcare Interoperability Resources (FHIR) is not assumed as a foundational data model in the design of the proposed framework. The structure and semantics of the medical record data are derived from national regulatory guidelines on EMR metadata, as specified in the Decree of the Minister of Health of the Republic of Indonesia No. HK.01.07/MENKES/1423/2022. In the prototype implementation, only a subset of the defined variables is utilized, as this subset is sufficient to demonstrate the proposed access control framework. Additional data elements can be incorporated without affecting the underlying governance design when required by future system extensions.

The proposed access control framework is designed to be data model-agnostic and does not rely on FHIR-specific authorization extensions or interfaces. Access policies, consent rules, and audit mechanisms are defined at the governance layer and enforced by integrating access control components with a DLT-based trust infrastructure. This separation of concerns allows the framework to remain applicable across different healthcare data representations while preserving patient sovereignty, cross-institution accountability, and tamper-evident auditing.

By explicitly decoupling data representation from access governance, the proposed approach addresses the limitations of interoperability-focused solutions that emphasize data exchange while neglecting governance challenges. The integration of a DLT-based governance layer complements existing healthcare data standards by providing verifiable access control and auditability in distributed healthcare ecosystems.

### 4.4. Mechanism of Account Management and Activation for Patients and Hospital Personnel

**Figure 4:**
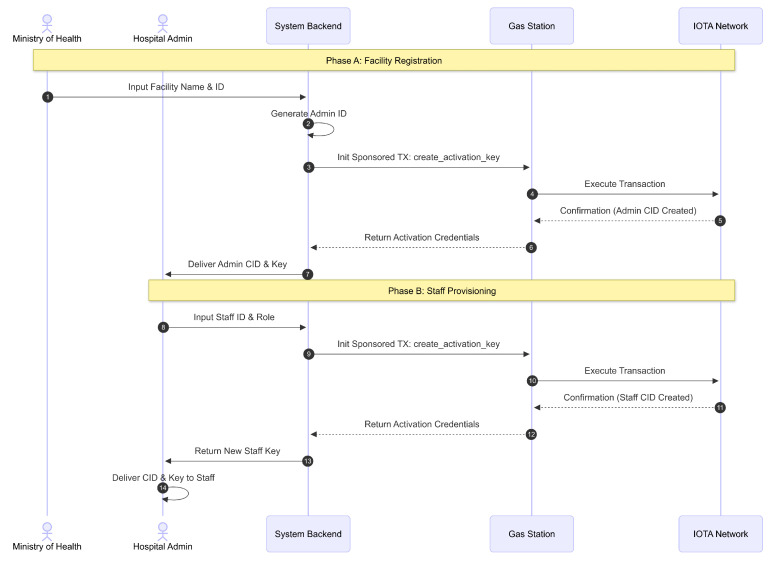
Mechanism for facility registration and hospital admin key generation.

In the proposed design, healthcare facility personnel account management begins with a controlled registration and activation process, as illustrated in Figure 4. The hospital admin for each healthcare facility must first receive the CID and activation key generated and issued by the Ministry of Health. This CID and activation key pair is strictly one-to-one and used to activate the personnel’s client application. The activation key can be used only once and thereafter serves as a cryptographic witness for each authorized action performed by the activated Client, providing secure proof of authorized access. The Ministry of Health initiates this process by entering the facility’s name and ID, after which the system automatically generates a fixed admin ID for the facility. This process involves a sponsored transaction relayed through a gas station server to the IOTA network, ensuring tamper-resistant registration records. Once the activation is successfully processed, the activation credentials are delivered. Subsequently, the hospital admin creates activation keys for additional hospital staff, such as medical and administrative personnel, by assigning IDs and roles. These keys are generated through a similar sponsored transaction mechanism and delivered to the respective staff members.

**Figure 5:**
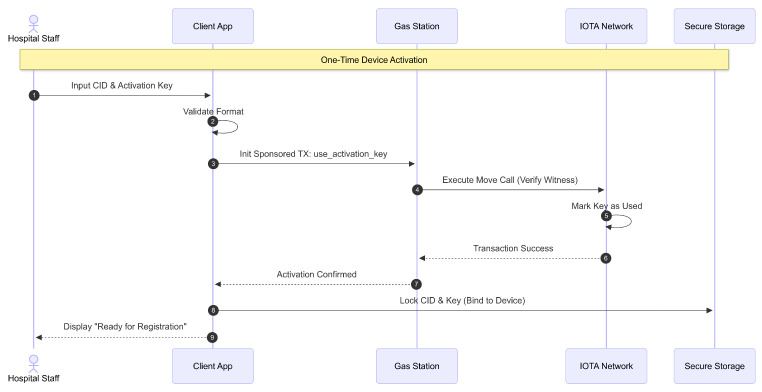
Client activation mechanism.

Figure 5 details the subsequent activation phase. Each staff member must activate their client application by inputting their CID and activation key, which validates their access and links their identity to the system’s access control. Activation is a one-time process per device installation, preserving the secure linkage of access rights.

**Figure 6:**
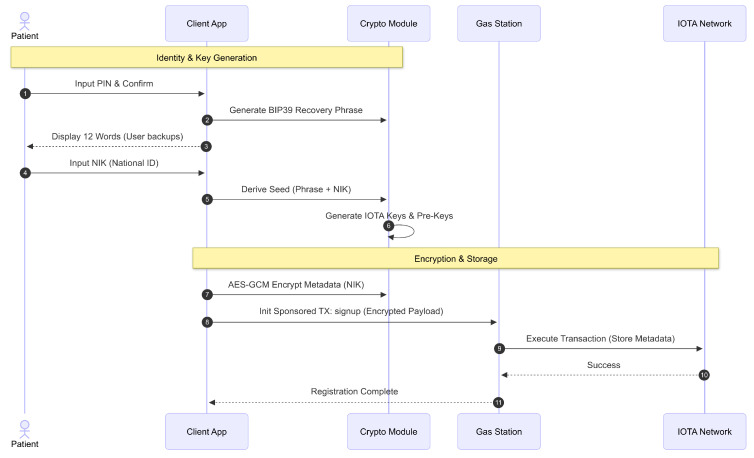
Patient and healthcare personnel account registration.

Following personnel activation, the system facilitates account registration for both patients and healthcare personnel with distinct but analogous procedures, detailed in Figure 6. Patients begin by choosing a secure six-digit PIN, which is validated along with a confirmation code. The system then generates a confidential 12-word recovery phrase based on the Bitcoin Improvement Proposal 39 (BIP39) standard, which the patient must confirm and securely store. This phrase is pivotal because it seeds the cryptographic keys used for data encryption and recovery. Patients subsequently provide their NIK. Using the recovery phrase and NIK as a passphrase, a cryptographic seed is derived to generate key pairs and addresses used in the IOTA distributed ledger. Sensitive private administrative data, initially limited to the NIK at registration, is encrypted using Advanced Encryption Standard in Galois/Counter Mode (AES-GCM) with randomly generated keys and nonces, which are themselves encrypted through PRE to safeguard against unauthorized access. All administrative metadata is then stored on the IOTA network via sponsored transactions, ensuring immutable, secure storage. Healthcare personnel undergo a similar registration process, but do not need to submit a NIK because they have a prior client activation. Instead, they register by entering a PIN and recovery phrase, with their assigned CID serving as a unique identifier that establishes equivalence to patient NIKs in the system. This mechanism ensures that both patient and staff identities are cryptographically secured and linked to their entries in the distributed ledger.

**Figure 7:**
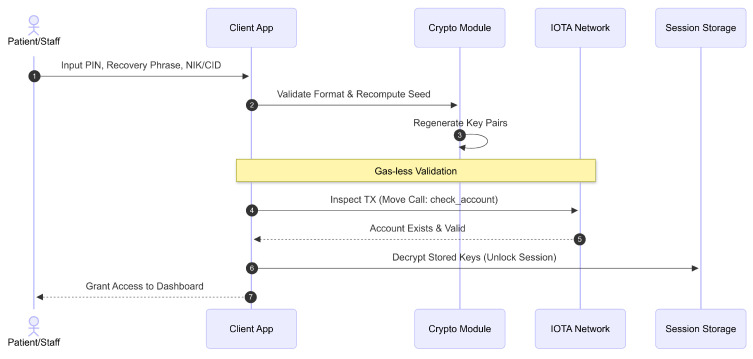
Sign in mechanism.

Once registered, patients and healthcare personnel access their accounts through a secure sign-in mechanism that emphasizes data protection and continuity, as shown in Figure 7. Patients must enter their PIN, confirmation PIN, previously issued recovery phrase, and NIK to decrypt their locally stored encrypted keys and gain access to their medical records. The system verifies the format of these credentials and recomputes the cryptographic seed to regenerate key pairs and addresses. It then queries the IOTA ledger to confirm the user’s account exists, without incurring transaction fees, using an inspection method. Upon successful validation, sensitive data—including encrypted keys and metadata—is securely stored on the Client’s device for use during the session. Healthcare personnel follow a parallel sign-in workflow that omits NIK input, relying instead on their CID assigned during client activation as their unique identifier. This consistent and secure authentication process ensures appropriate role-based access management, facilitating seamless access to EMR while maintaining strong cryptographic protections and compliance with healthcare data privacy regulations.

Next, the workflow mechanism related to the patient’s EMR consists of two parts: one for administrative personnel and another for medical personnel. However, only the medical personnel’s workflow will be presented here due to space constraints, as the processes for both roles are largely similar.

### 4.5. Mechanism for Granting Medical Personnel Access to Read Administrative and Clinical Data and Edit Patient Clinical Data

**Figure 8:**
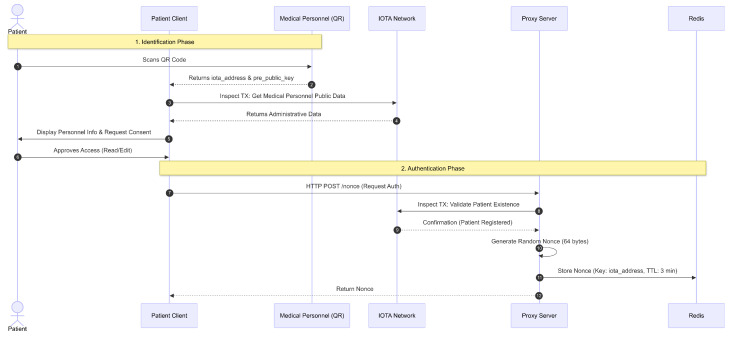
Identification and authentication mechanism for granting medical personnel access to read administrative and clinical data and edit patient clinical data.

**Figure 9:**
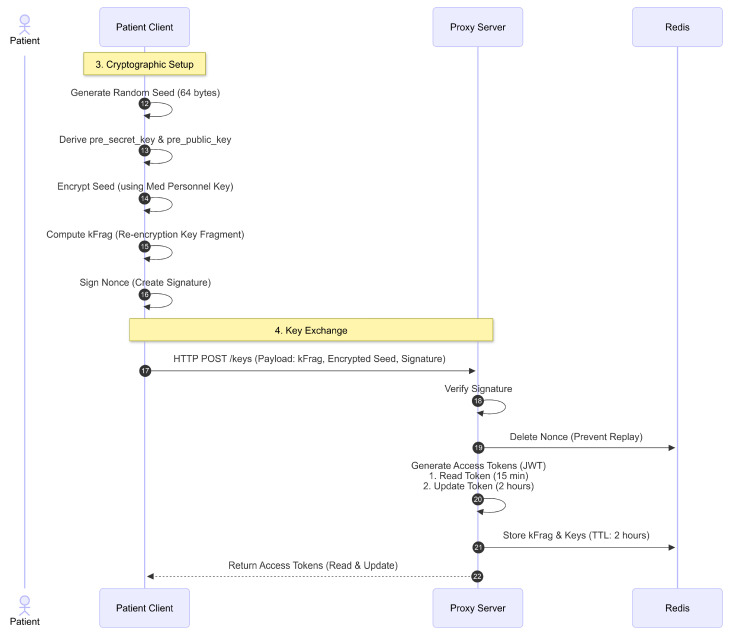
Key generation and token exchange mechanism for granting medical personnel access to read administrative and clinical data and edit patient clinical data.

**Figure 10:**
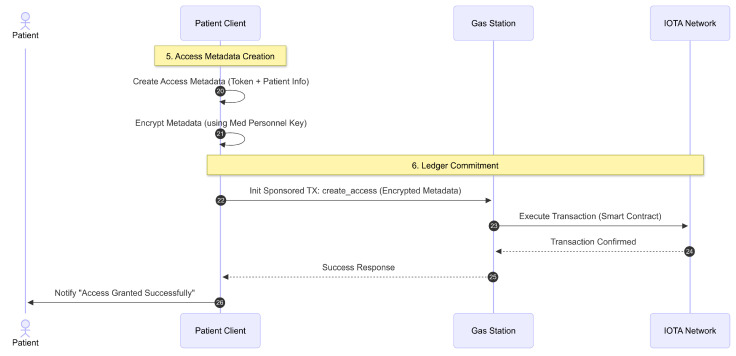
Ledger access mechanism for granting medical personnel access to read administrative and clinical data and edit patient clinical data.

The mechanism for granting medical personnel access to read and edit patients’ EMR is illustrated in Figure 8, Figure 9 and Figure 10. The identification and authentication phase is detailed in Figure 8. First, the patient must scan the QR code owned by the medical personnel. The QR code contains the iota_address and pre_public_key of the medical personnel. After obtaining the iota_address and pre_public_key from the medical personnel, these data are used to retrieve the medical personnel’s public administrative data stored on the IOTA network. This is to ensure that the patient can confirm the QR code indeed belongs to medical personnel from the corresponding healthcare facility.

The public administrative data obtained from the IOTA network is then displayed on the patient’s Client. At this stage, the patient will also see a confirmation dialogue. The confirmation dialogue displays public administrative data for the medical personnel and a question about granting read, write, and edit access to them. The patient can then approve or deny this access.

If the patient approves the access, the Client will make an HTTP POST request to the PRE server at POST/nonce. This request seeks a nonce. Next, the PRE server validates whether the patient indeed made the request. Validation is performed by executing a Move call on the IOTA network in inspecttransaction mode. The Move call invokes a function to check whether the provided iota_address is registered as a patient.

Once it is confirmed that the request is from the patient, the PRE server generates a 64-byte random nonce, which is used as an authentication component for the patient in the next request. The nonce is then stored in Redis as a HEX string key with the iota_address value. The nonce stored in Redis has a Time To Live (TTL) of 3 min and is single-use to prevent replay attacks. Additionally, the PRE server returns the nonce as the response to the request.

The cryptographic setup and token exchange process are shown in Figure 9. After receiving the nonce from the PRE server, the patient client generates a random 64-byte seed. This seed is then used to generate new pre_secret_key and pre_public_key (med_pre_secret_key and med_pre_public_key). These keys serve as the private and public keys in the PRE mechanism, enabling medical personnel to decrypt the aes_key and aes_nonce from the patient’s clinical and administrative data.

The first half (32 bytes) of the seed is then encrypted using the medical personnel’s pre_public_key (medical_personnel_pre_public_key). The result of this encryption process is enc_first_half_seed and first_half_seed_capsule. Then, the Client generates random signer_pre_sec_ret_key and signer_pre_public_key keys, which are also used in the PRE process. The client computes kfrag using the patient’s pre_public_key, adm_pre_public_key, and signer_pre_secret_key. kfrag is a key component of the PRE mechanism that medical personnel will later use to decrypt the patient’s clinical and administrative data using the aes_key and aes_nonce.

The Client then signs the nonce received. This signature serves as proof that the nonce belongs to the patient and will be verified by the PRE server. Next, the Client sends a POST/keys request to the PRE server. The payload sent by the patient client includes enc_first_half_seed, the medical personnel’s iota_address, kfrag, med_pre_public_key, first_half_seed_capsule, the patient’s iota_address, the patient’s pre_public_key, the signature, and signer_pre_public_key.

The PRE server verifies the signature. If valid, the previously stored nonce in Redis is automatically deleted. Then, the Proxy creates an access token in JSON Web Token (JWT) format. Unlike the access tokens for administrative personnel, for medical personnel, the Proxy generates two access tokens: one for read access and one for edit access. The difference lies in their expiration times: 15 min for read access and 2 h for edit access.

Both access tokens contain claims such as the role set to MedicalPersonnel and the access type (purpose), either Read for read access or Update for edit access. Other payload data sent by the patient client—enc_first_half_seed, kfrag, med_pre_public_key, first_half_seed_capsule, pre_public_key, and signer_pre_public_key—are stored in Redis with a TTL of 2 h. The two access tokens are then returned as the response.

Figure 10 demonstrates the final phase of committing the access grants to the ledger. The patient client creates two metadata access entries for the medical personnel. These metadata access entries are of types Read and Update, containing the patient’s name, patient’s iota_address, the corresponding access token, and the patient’s pre_public_key. These metadata access entries are then encrypted using the medical personnel’s pre_public_key so they can be decrypted with the corresponding pre_secret_key. Finally, the patient client stores both metadata access entries on the IOTA network by initiating a sponsored transaction (create_access) through the gas station server.

The gas station server continues executing the transaction on IOTA. After successful execution, medical personnel can access the patient’s administrative and clinical data within the specified time frame.

### 4.6. Mechanism of Accessing Patient Administrative and Clinical Data by Medical Personnel

**Figure 11:**
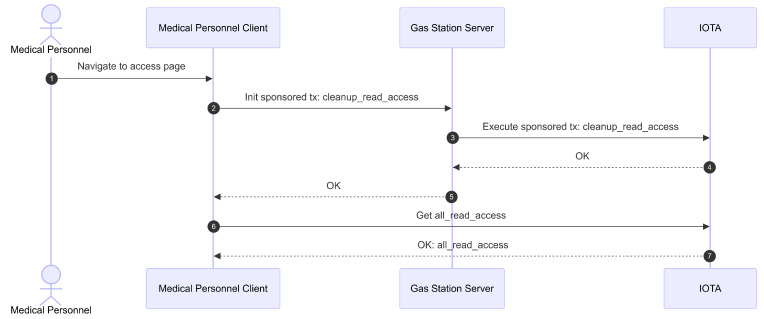
Initialization and access listing mechanism for accessing patient administrative and clinical data by medical personnel.

**Figure 12:**
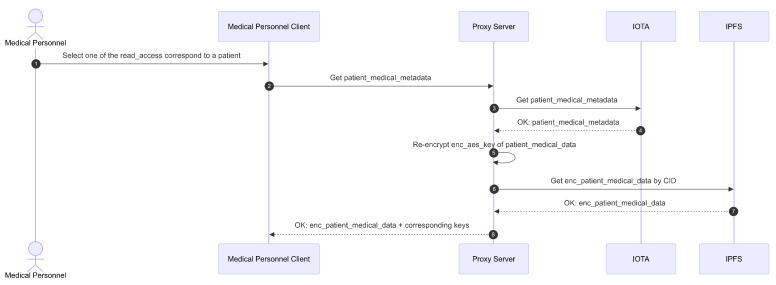
Metadata retrieval and proxy re-encryption mechanism for accessing patient administrative and clinical data by medical personnel.

**Figure 13:**
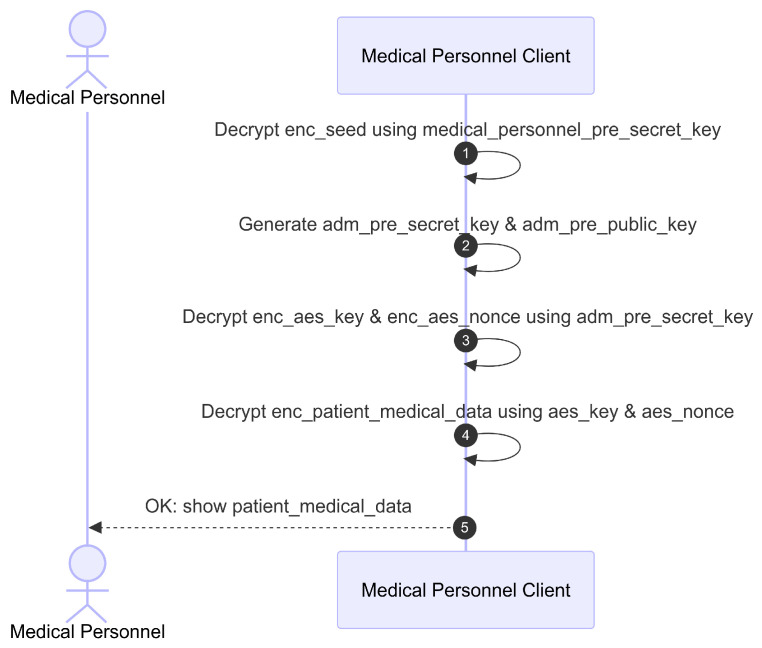
Client-side decryption and data rendering mechanism for accessing patient administrative and clinical data by medical personnel.

The mechanism for accessing patients’ administrative and clinical data by medical personnel is illustrated in Figure 11, Figure 12 and Figure 13. The initial phase of the read access mechanism is illustrated in Figure 11. First, the medical personnel navigate to the access page on the client application. The Client then initiates a sponsored transaction cleanup_read_access through the gas station server. The gas station server then executes this transaction on the IOTA network. This transaction is intended to clean up any expired read access entries.

After the transaction is successfully executed, the Client queries all read accesses currently held by the medical personnel. This query is performed via a Move call to the IOTA network in inspect transaction mode. The Move call triggers a function that returns all read access permissions held by medical personnel as access metadata (SubSection 4.5). The names of the patients associated with these access entries are displayed as a list on the access page.

The second phase, as shown in Figure 12, involves retrieving specific patient records and performing re-encryption. After the first phase, the medical personnel can then select a patient from this list. Next, the Client sends a GET/medical-record request to the PRE server, including the patient’s iota_address and an index parameter. The index serves as the ID for the patient’s EMR entry, necessary because multiple entries can exist and only one can be displayed at a time. The access token obtained from the metadata is used as a Bearer token in the authentication header. The Proxy validates this access token; if it is valid, unexpired, and contains the correct claims, the Proxy queries the IOTA network via Move call (inspect transaction mode) to retrieve the patient’s administrative and clinical metadata.

The function invoked by the Move call verifies that the medical personnel have valid access rights to the patient’s administrative and clinical data. This validation uses the IOTA network’s Clock feature. After obtaining the metadata, the Proxy re-encrypts enc_aes_key_nonce using the metadata. The re-encryption process involves the capsule from the metadata (aes_key_nonce_capsule) and the kfrag stored in Redis, resulting in a cfrag.

Once re-encryption is complete, the Proxy retrieves the patient’s enc_medical_data from IPFS using the CID obtained from the clinical metadata. The proxy then sends a response containing cfrag, med_pre_public_key, first_half_seed_capsule, enc_first_half_seed, enc_private___administrative_data, enc_medical_data, enc_aes_key_nonce (for administrative data), enc_aes_key_nonce (for clinical data), the patient’s pre_public_key, aes_key_nonce_capsule, and signer_pre_public_key.

The final phase, illustrated in Figure 13, describes the local decryption process on the client side. The Client decrypts the enc_first_half_seed using the medical personnel’s pre_public_key. The resulting 32-byte seed is used to generate med_pre_secret_key and med_pre_public_key. The Client decrypts the enc_aes_key_nonce using the med_pre_secret_key. The decrypted aes_key and aes_nonce are then used to decrypt the patient’s enc_private_administrative_data and enc_medical_data. Finally, the Client displays the patient’s administrative and clinical data.

### 4.7. Mechanism for Creating New EMR Entries by Medical Personnel

**Figure 14:**
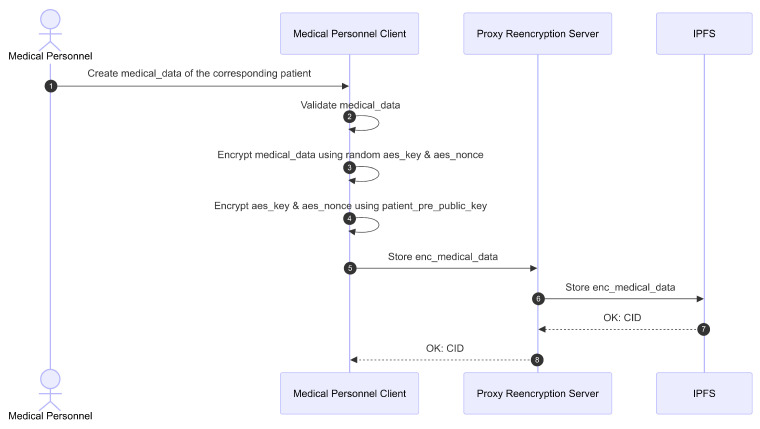
Workflow of the editing process for EMR entries by medical personnel (Part 1).

**Figure 15:**
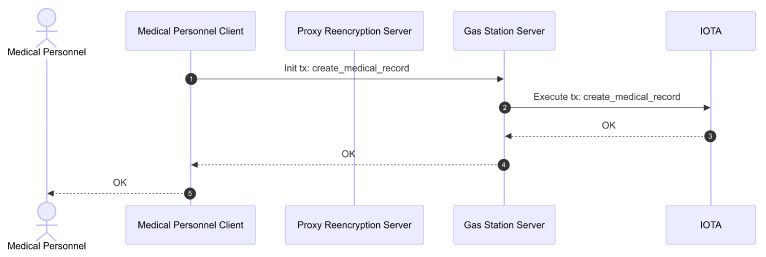
Workflow of the editing process for EMR entries by medical personnel (Part 2).

After examination, medical personnel can create a new EMR entry for the respective patient. The process for creating a new entry is illustrated in Figure 14 and Figure 15. The initial stage of this process is illustrated in Figure 14. First, the medical personnel must input the relevant clinical data. The Client then validates this data. Once confirmed valid, the patient’s clinical data is encrypted using the AES-GCM algorithm. The aes_key and aes_nonce required for AES-GCM are randomly generated by the Client. This encryption produces enc_medical_data. The generated aes_key and aes_nonce are then encrypted using the patient’s pre_public_key, resulting in enc_aes_key_nonce and capsule.

Next, the Client sends a POST/medical-record request to the PRE server. The request payload includes the patient’s iota_address, enc_medical_data, enc_aes_key_nonce, and capsule. The Proxy stores the enc_medical_data on IPFS and obtains a CID for the stored data. The CID, enc_aes_key_nonce, the capsule, and the operation timestamp (created_at) are then saved on the IOTA network as the patient’s clinical metadata (medical_metadata).

The second stage, focusing on ledger registration, is illustrated in Figure 15. The CID obtained from IPFS, along with the enc_aes_key_nonce, capsule, and a timestamp (created_at), are assembled into the patient’s clinical metadata (medical_metadata). Storing on the IOTA network is done by initiating a sponsored transaction (sponsored tx: create_medical_record) via the gas station server. The gas station server then continues executing the transaction on IOTA. Once the transaction is successfully executed, the new EMR entry is added. Creating a new EMR entry for a patient can be done only once per Update access granted by the patient. After creating the new entry, medical personnel can only edit it within the specified time window.

### 4.8. Mechanism for Editing New EMR Entries by Medical Personnel

After creating a new EMR entry, medical personnel can edit it for up to 2 h after the patient grants access. The editing mechanism is similar to the creation process shown in Figure 14 and Figure 15, with the main difference being the updated clinical data.

First, medical personnel input the relevant clinical data. The Client then validates this data. Once confirmed valid, the edited clinical data is encrypted using the AES-GCM algorithm. The aes_key and aes_nonce required by AES-GCM are randomly generated by the Client. This encryption produces enc_medical_data. The generated aes_key and aes_nonce are then encrypted using the patient’s pre_public_key, resulting in enc_aes_key_nonce and capsule.

Next, the Client sends a PUT/medical-record request to the PRE server. The payload includes the patient’s iota_address, enc_medical_data, enc_aes_key_nonce, and capsule. The Proxy stores the enc_medical_data on IPFS and obtains the corresponding CID. The CID, along with enc_aes_key_nonce, capsule, and the timestamp of the operation (created_at), is then saved to the IOTA network as the patient’s updated clinical metadata (medical_metadata).

Storage on the IOTA network is provided by initiating a sponsored transaction (create_medical_record) via the gas station server. The gas station server then executes the transaction on IOTA. Once the transaction is successfully executed, the EMR entry is considered successfully edited. Medical personnel can make unlimited edits to the EMR data within 2 h of the patient granting update access.

### 4.9. Mechanism for Revoking Access to EMR Data

**Figure 16:**
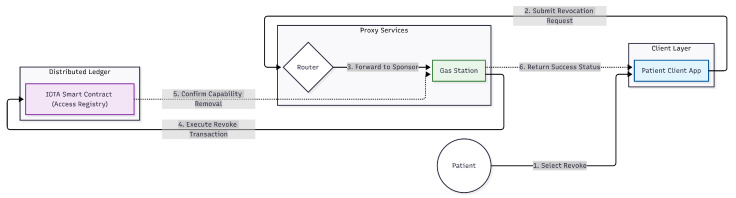
Process flow of revoking patient access rights to EMR data via the sponsored transaction mechanism.

The process of revoking access to a patient’s EMR data can be seen in Figure 16. This process is carried out by the patient, who first selects the access entry they wish to revoke. Then, the Ministry of Health client initiates a sponsored transaction (revoke_access) through the gas station server. The gas station server then executes this transaction on the IOTA network. Once the transaction is successfully executed, the access entry is deleted, and access to the EMR data is effectively revoked.

## 5. Implementation

In this section, the focus will be on implementing the system’s two main components: the Smart Contract and the Proxy. The client component will not be discussed in detail as it mainly involves the user interface (UI) implementation and is not the primary focus of this research.

### 5.1. Client

The clients for patients, healthcare personnel, and the Ministry of Health are implemented using the Tauri framework, which combines the Rust programming language with SvelteKit, built on Typescript. The patient client is developed as a mobile application, while the clients for healthcare personnel and the Ministry of Health are designed as desktop applications. Each client consists of multiple pages to facilitate interaction with the system’s features. However, the client implementation is not the primary focus of this research; it serves mainly to demonstrate the functional capabilities of the overall system.

### 5.2. PRE Server

The PRE server is implemented in Rust using the Axum web framework. The proxy provides several endpoints accessible by both patient clients and healthcare personnel clients.

The POST/nonce endpoint generates a 64-byte random nonce for authenticating patient clients when they make requests to the proxy. After validating the request and confirming the patient’s registration via a Move call to the IOTA smart contract, the nonce is stored in Redis with a 3-min expiration and returned in the response.

The POST/keys endpoint stores data necessary for PRE and generates JWT access tokens for healthcare personnel. After validating the request and verifying the patient’s signature, the proxy identifies the personnel’s role through a Move call and issues access tokens with durations based on role and access type. Corresponding patient data is stored in Redis with a TTL equal to the longest token duration.

The GET/medical-record endpoint allows medical personnel with valid read access tokens to retrieve a patient’s administrative and clinical metadata. The proxy validates the token, fetches the data from Redis and IOTA via Move calls, and performs PRE to generate a cfrag for decryption. Encrypted clinical data is also retrieved from IPFS.

The POST/medical-record endpoint enables medical personnel with update access tokens to create new medical record entries. The proxy validates the token and request, stores encrypted clinical data on IPFS, obtains a CID, and records metadata and timestamps on IOTA through sponsored transactions.

The PUT/medical-record endpoint allows medical personnel to edit existing medical record entries within the same visit, following a process similar to the create endpoint but calling an update method on the smart contract.

The GET/medical-record/update endpoint lets medical personnel retrieve updated administrative and clinical metadata for editing, validates update access tokens, and uses PRE to access encrypted data, similar to the read endpoint.

Finally, the GET/administrative endpoint enables hospital admins and administrative personnel with read access tokens to fetch administrative metadata. The proxy verifies access, retrieves data from Redis and IOTA, and applies PRE to decrypt the data. Together, these endpoints form the core of DecMed’s proxy server functionality, securely managing access control, data retrieval, encryption, and updating operations.

### 5.3. Smart Contract

The smart contract is implemented using the Move programming language. Move-based smart contracts run on IOTA’s Layer 1 (L1) network, offering performance advantages and lower gas fees. In IOTA Move, smart contracts are implemented as packages composed of multiple modules, each containing contract code. Before execution, the smart contract package must be published. The published part consists of one or more modules within the package.

The methods implemented within these modules include read-only methods, which are designed to retrieve or view data from the ledger. It is important to note that executing these read-only methods does not incur any gas fees.

The system’s smart contract is implemented as a package named DecMed. The DecMed package is divided into several modules. Broadly, the DecMed package consists of seven main modules, as follows:

The decmed::patient module contains various methods that patients can execute. A complete list of the methods implemented in this module is presented in Table 5.

The decmed::admin module includes methods that the Ministry of Health can execute. The methods implemented within this module are listed in Table 6.

The decmed::hospital_personnel module provides methods accessible to healthcare facility personnel, including hospital administrators, medical personnel, and administrative personnel. The full list of methods in this module is available in Table 7.

The decmed::proxy module contains methods executed by the proxy server. A detailed list of these methods is provided in Table 8.

The decmed::shared module contains utility methods for performing certain computations. These methods are accessible and can be used by other modules. Additionally, this module defines the capabilities of the Ministry of Health and its proxy. The implemented methods in this module are listed in Table 9.

Modules prefixed with decmed::std_struct_* represent data structures of objects stored on the IOTA network. Among these, the decmed::std_struct_hospital_personnel_access_data module plays a crucial role in the RME data access control mechanism. This module defines the data structure representing the system’s CapBAC. The structure is shown in Listing 1 and includes attributes such as the types of accessed data (access_data_types), access duration (exp), metadata (metadata), and the index of the most recently added RME (medical_metadata_index).

Similarly, modules prefixed with decmed::std_enum_* represent enums that are part of the data structure for objects stored on IOTA. Several enums are implemented, such as GlobalAdminCap for representing the Ministry of Health’s capability and ProxyCapEnum for representing the PRE server’s capability.

## 6. Evaluation

The evaluation focuses on illegal access scenarios targeting electronic medical record (EMR) data. These scenarios are not defined as arbitrary use cases, but are derived from a threat modelling process in which EMR data is treated as the primary protected asset. Scenario selection follows the STRIDE threat modelling framework, with particular emphasis on threats related to Information Disclosure, Tampering, and Elevation of Privilege, which are commonly associated with unauthorized access in healthcare information systems.

A smart contract ultimately controls all access to EMR data; therefore, the evaluation focuses on its behaviour and enforcement logic. The smart contract is evaluated using the IOTA CLI and the built-in unit testing framework provided by IOTA Move. Each illegal access scenario is identified using the format T-SC-ILLXX, where XX is a two-digit number. The complete list of evaluated scenarios is presented in Table 10.

As previously explained, the smart contract component is evaluated through unit testing, with one evaluation method per scenario. These methods follow the naming format test_ill_xx, where xx is a two-digit number corresponding to the scenario evaluation ID.

At the beginning of each method, a setup is performed. This setup is further divided into three different methods: setup_shared_objects, setup_data, and add_medical_record. The setup_shared_objects method is used to mock the shared objects required as arguments for the evaluation methods. The setup_data method is used to mock the accounts of each actor involved. The add_medical_record method adds an EMR entry to the previously created mocked patient account. After the setup is completed, the necessary methods for each scenario are invoked.

In the EInvalidAccessType expected result scenario (T-SC-ILL01), an administrative personnel member attempts to read the clinical section of a patient’s EMR. This action is carried out via the get_medical_record_test method, which retrieves both the administrative and clinical sections of the EMR. Since the requested access type is not valid for the personnel’s permission, the method returns the EInvalidAccessTyp error.

In the EAccessExpired expected result scenarios (T-SC-ILL02, T-SC-ILL04, T-SC-ILL06, T-SC-ILL09), multiple cases demonstrate access attempts after the allowed time limit has passed. First, an administrative user attempts to read the administrative section of a patient’s EMR, with the IOTA network time set to 10 min after access was granted—exceeding the 5-min limit. The get_administrative_data_test method processes the request and returns the EAccessExpired error. Next, a medical personnel member attempts to read both the administrative and clinical sections of a patient’s EMR, with the IOTA network time set to 20 min after access, surpassing the 15-min limit. The request is made through the get_medical_record_test method, which returns the same EAccessExpired error. Another case involves medical personnel attempting to add a new EMR entry after 3 h, exceeding the 2-h limit for write operations. This attempt, made using the create_medical_record_test method, also triggers the EAccessExpired error. Finally, medical personnel try to edit an EMR entry 3 h after access was granted, again surpassing the 2-h limit. The update_medical_record_test method is used, and it returns the EAccessExpired error.

In the EAccessNotFound expected result scenarios (T-SC-ILL03, T-SC-ILL05, T-SC-ILL08, T-SC-ILL10), access is attempted without prior authorization from the patient. First, an administrative personnel tries to read the administrative section of a patient’s EMR using the get_administrative_data_test method. However, the requested IOTA address does not match the one for which access was granted, resulting in the EAccessNotFound error. Similarly, medical personnel attempt to read both the administrative and clinical sections of a patient’s EMR via the get_medical_record_test method, but since no access was granted for the target patient, the system returns EAccessNotFound. In another case, medical personnel try to add a new EMR entry for a patient without granted access using the create_medical_record_test method, which leads to the same error. Lastly, medical personnel attempt to edit an EMR entry for a patient without granted access through the update_medical_record_test method, which again results in the EAccessNotFound error.

In the EMedicalRecordCreationLimit expected result scenario (T-SC-ILL07), medical personnel create a new EMR entry during an active access period and then attempt to create another entry within the same period. Both actions are carried out using the create_medical_record_test method. The second attempt triggers the EMedicalRecordCreationLimit error because the system enforces a limit of 1 EMR creation per access period.

In the T-SC-ILL11 scenario, medical personnel attempt to perform a write operation by editing an EMR entry created by other medical personnel. This scenario is not implemented as test code like the previous scenarios because, to perform a write operation (such as editing), the IOTAaddress of the medical personnel who will perform the edit must be specified from the beginning. The smart contract then automatically checks the access rights associated with that address, so edits can only be made to EMR data created by the same medical personnel.

## 7. Results and Discussion

This section discusses the results from implementing and evaluating the proposed DecMed framework. Rather than focusing solely on functional correctness, the discussion emphasizes how the results validate the proposed access governance design and its alignment with the stated research questions.

### 7.1. RQ1: Patient-Centric Access Control Design for Cross-Facility EMR Integration

**Figure 17:**
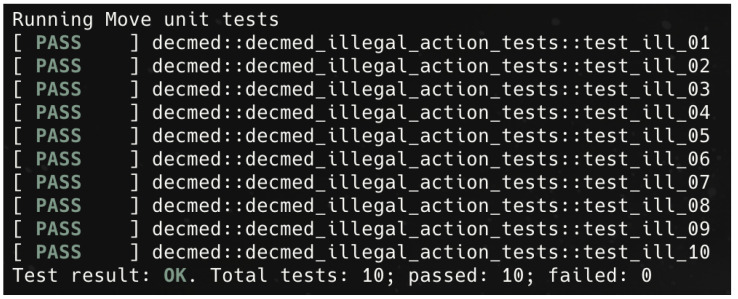
Summary of smart contract component testing results.

The evaluation of the smart contract component under illegal EMR access scenarios, as described in Section 6, demonstrates that the proposed access control design consistently enforces patient-centric authorization policies across all tested cases. All test scenarios produced the expected outputs, indicating that access permissions are validated against patient-issued consent, access scope, and temporal constraints. A summarized overview of the evaluation results is presented in Figure 17.

The structured scenario-based evaluation enables validation of access control behavior under different violation conditions. For example, access attempts that exceeded the authorized time window or violated access type constraints consistently resulted in EAccessExpired and EInvalidAccessType errors. Similarly, unauthorized access attempts without valid consent resulted in EAccessNotFound responses, confirming that access rights are checked before any data operation.

These results indicate that access enforcement is not institution-centric but instead anchored to patient-defined consent and policy constraints. By ensuring that access rights are explicitly granted, time-bound, and revocable, the framework preserves patient data sovereignty while enabling controlled access across healthcare facilities. In addition, restrictions on EMR creation and modification, such as limiting record updates to the originating medical personnel, contribute to accountability and traceability of medical data handling.

However, the current evaluation scope does not yet cover all healthcare delivery contexts defined in Indonesian regulations, such as inpatient or emergency scenarios where patients may be unable to provide direct consent. These cases require additional mechanisms, including emergency access or delegated consent models, which are identified as future extensions.

Overall, the results for RQ1 demonstrate that a patient-centric access control framework can be designed to support cross-facility EMR integration while preserving data sovereignty and auditability through explicit consent enforcement and policy validation.

### 7.2. RQ2: Role of Distributed Ledger Technology in Enforcing Access Governance

Beyond implementation concerns, the use of Distributed Ledger Technology (DLT) in the proposed framework plays a fundamental role in shaping how access governance is enforced and verified across independent healthcare institutions. Rather than acting merely as a data storage or transaction layer, DLT functions as a shared governance infrastructure that embeds access control rules and auditability into the system’s core.

In the DecMed framework, access validation logic is anchored in on-chain smart contracts, ensuring that authorization decisions are evaluated consistently regardless of the originating healthcare facility. This design enables access policies, consent constraints, and expiration rules to be enforced through immutable state transitions, reducing reliance on institution-specific enforcement mechanisms. As a result, access governance becomes verifiable and independently auditable by participating entities, rather than relying on a single organizational authority.

The integration of off-chain services, such as the proxy server and PRE mechanisms, complements this governance model by enabling practical data handling while deferring authorization decisions to the ledger. By separating data storage from access validation, the framework maintains scalability and privacy while preserving the ledger’s role as the authoritative source for access governance.

From a governance perspective, the results demonstrate that DLT enables a shift from institution-centric control toward policy-driven enforcement supported by algorithmic verification. Access events, consent grants, and policy violations are recorded in a tamper-evident manner, providing a reliable audit trail that supports accountability across organizational boundaries. This property is particularly relevant in national-scale healthcare ecosystems, where institutions operate at comparable levels of authority, and no single entity can be assumed to serve as the sole trusted controller.

Overall, the results for RQ2 show that DLT plays a critical role in enforcing and verifying access governance by providing a shared, verifiable, and tamper-evident governance layer. Rather than simply hosting access control logic, the ledger actively shapes how access policies are enforced and trusted in cross-institution EMR environments.

### 7.3. Limitations

The proposed framework is evaluated as a governance validation prototype and does not yet address user experience, performance benchmarking, or large-scale deployment considerations. The system’s reliance on distributed infrastructure makes it sensitive to network stability, as synchronization delays among IOTA nodes and off-chain storage services can affect responsiveness in low-bandwidth environments.

Furthermore, the evaluation focuses on the correctness of access governance rather than usability or clinical workflow integration. Direct involvement of patients and healthcare professionals would require ethical approval and deployment within operational healthcare systems, which are beyond the scope of this study. These aspects are identified as important directions for future work, including user-centred evaluation and pilot deployment in real-world healthcare settings.

## 8. Future Work

This study opens several avenues for future research to improve the proposed EMR access control system further. First, future development should focus on supporting more comprehensive healthcare service mechanisms. For instance, implementing an emergency access delegation feature would allow authorized medical personnel to access a patient’s EMR when the patient, as the data owner, is unable to provide direct consent. This mechanism would ensure timely medical intervention while preserving security and regulatory compliance. Second, the system should be extended to support broader access delegation mechanisms. This includes granting controlled access to external parties such as health insurance providers or researchers conducting health-related studies, enabling the EMR system to be more adaptable to diverse healthcare ecosystem needs. Third, a more comprehensive account management system should be designed. This could include a “forgot recovery phrase” feature and multi-device login, allowing patients to securely access their accounts from multiple devices while maintaining strong security guarantees. Fourth, future work should integrate a built-in mechanism for distributing activation keys and healthcare personnel CIDs directly within the system, streamlining onboarding and identity verification processes. Finally, a more comprehensive registration mechanism for healthcare facilities and their administrators should be developed. This could involve enabling healthcare facilities to self-register and manage multiple administrators, thereby enhancing administrative flexibility and scalability of the EMR network.

## 9. Conclusions

In this study, an access control mechanism within the framework of an EMR management system was successfully designed and implemented using IOTA DLT. The proposed system, named DecMed, fulfils the objectives defined at the outset of the work, aiming to enhance the security of EMR data, particularly in terms of confidentiality and integrity, in compliance with the Regulation of the Minister of Health of the Republic of Indonesia No. 24 of 2022 concerning Medical Records.

DecMed’s access control mechanism ensures that any access to a patient’s EMR can only occur after the patient has granted explicit consent. It further allows fine-grained control by limiting both the duration of access and the specific portions of the EMR that may be shared, aligning with the requirements of healthcare service delivery in Indonesia. The framework also incorporates account management features for both patients and healthcare facility personnel, tightly integrated with the access control mechanism.

Comprehensive evaluations confirm that the system operates as intended and meets all predefined requirements. These results demonstrate that the proposed framework effectively strengthens EMR data security while remaining adaptable to the operational and regulatory context of healthcare in Indonesia.

## Figures and Tables

**Table 1:** Comparison of alternative access controls.

Requirement	RBAC	ABAC	CapBAC
Granularity	✗	✓	✓
Interoperability	✗	✗	✓
Delegation	✗	✗	✓
Automatic Revocation	✗	✗	✓
Scalability	✗	✓	✓
Distributed nature	✗	✗	✓
Ease of use	✓	✗	✓

**Table 2:** Comparison of alternative DLTs.

Criteria	Hyperledger Fabric	IOTA
TPS	∼6000 [38]	50,000+ [35]
Sponsored transactions	– (No gas fee)	Easy (official tooling)
Time to finality (ms)	–	∼500 [33]
Smart contract language	JavaScript, Java, or Go	Move on L1. Solidity on L2
Consensus	Raft	dPoS with Mysticeti
Network structure	Blockchain	DAG

**Table 3:** Actors and their privileges.

Actor	Privileges
Patient	Read their own EMR data,
	Grant read and write access to their own EMR data,
	Revoke read and write access to their own EMR data,
	View access logs of their own EMR data.
Medical personnel	Read patient EMR data (administrative and clinical sections) after being granted access by the patient,
	Add new patient EMR data (clinical section only) after being granted access by the patient,
	Edit newly added EMR data (clinical section only) from the same visit after being granted access by the patient.
Administrative personnel	Read patient EMR data (administrative section only) after being granted access by the patient.
Healthcare facility admin	Add healthcare facility personnel,
	Generate activation keys for healthcare facility personnel.
Ministry of Health	Add healthcare facilities along with their respective admin,
	Generate activation keys for healthcare facility admins.

**Table 4:** Client Subsystem and Actor Hierarchy Mapping.

Actor Group	Specific Role	Client App (Platform)	Description/Features
Patient	Patient	Patient Client App (Mobile)	Designed for portability; leverages native mobile features (e.g., biometric sensors, cameras) for identity verification.
Ministry	Ministry	Ministry Client App (Desktop)	Allows regulatory oversight and high-level auditing.
Hospital Personnel	Medical Personnel	Hospital Client App (Desktop)	Optimized for clinical workflows. Responsible for creating and accessing medical records.
	Administrative Personnel	Hospital Client App (Desktop)	Responsible for patient registration and billing data.
	Hospital Admin	Hospital Client App (Desktop)	Responsible for managing institutional keys and personnel access.

**Table 5:** The list of methods in the decmed::patient module.

Method Name	Read Only	Description
create_access	×	Used to create an access entry (*capability*) granted to healthcare facility personnel
is_account_registered	✓	Used to check whether an IOTA address already has a registered account on IOTA
signup	×	Used to register a patient account
get_account_info	✓	Used to retrieve patient account information
get_account_state	✓	Used to obtain the patient account’s state
get_hospital_personnel_info	✓	Used to get public administrative data of healthcare personnel
get_access_log	✓	Used to retrieve access logs for RME data
get_medical_record	✓	Used to get details of a specific RME entry
get_medical_records	✓	Used to get a list of RME entries owned by a patient
revoke_access	×	Used to revoke previously granted access
update_administrative_metadata	×	Used to edit the administrative part of a patient’s RME data

**Table 6:** The list of methods in the decmed::admin module.

Method Name	Read Only	Description
create_activation_key	×	Used to create an activation key for hospital admin and simultaneously register the hospital in the system
get_hospitals	✓	Used to retrieve the list of registered hospitals
update_activation_key	×	Used to edit the activation key of a registered hospital admin

**Table 7:** The list of methods in the decmed::hospital_personnel module.

Method Name	Read Only	Description
cleanup_read_access	×	Used to remove expired read access entries
cleanup_update_access	×	Used to remove expired update access entries
create_activation_key	×	Used to create a new activation key for other healthcare personnel
get_account_info	✓	Used to retrieve healthcare personnel account information
get_account_state	✓	Used to obtain the healthcare personnel account’s state
get_hospital_personnels	✓	Used by hospital admin to retrieve the list of personnel in their facility
get_read_access	✓	Used to retrieve the list of granted read access
get_update_access	✓	Used to retrieve the list of granted update/write access
is_account_registered	✓	Used to check if a healthcare personnel ID is already registered
signup	×	Used to register a healthcare personnel account
update_account_activation_key	×	Used by hospital admin to edit the activation key of registered personnel
update_administrative_metadata	×	Used to edit the administrative data of healthcare personnel
use_activation_key	×	Used to activate the healthcare personnel client

**Table 8:** The list of methods in the decmed::proxy module.

Method Name	Read Only	Description
create_capability	×	Used by the Ministry of Health to register the proxy
create_medical_record	×	Used to create a new RME entry
get_administrative_data	×	Used to retrieve the administrative part of a patient’s RME
get_hospital_personnel_role	×	Used to retrieve the role of a healthcare personnel
get_medical_record	×	Used to retrieve both administrative and clinical parts of a patient’s RME
get_medical_record_update	×	Used to retrieve administrative and clinical parts during the RME edit process
is_patient_registered	✓	Used to check if an IOTA address is registered as a patient
update_medical_record	×	Used to edit an RME entry

**Table 9:** The list of methods in the decmed::shared module.

Method Name	Read Only	Description
encode_hospital_id	✓	Used to encode the hospital ID
encode_hospital_personnel_id	✓	Used to encode the healthcare personnel ID
encode_patient_id	✓	Used to encode the patient ID
transfer_proxy_cap	×	Used to transfer ProxyCapability
transfer_global_admin_cap	×	Used to transfer GlobalAdminCapability

**Table 10:** Illegal access scenarios.

ID	Evaluation Scenario	Expected Return
T-SC-ILL01	Medical record staff attempts to read the clinical section of a patient’s EMR	EInvalidAccessType
T-SC-ILL02	Medical record staff attempts to read the administrative section of a patient’s EMR after the allowed time has expired	EAccessExpired
T-SC-ILL03	Medical record staff attempts to read the administrative section of a patient’s EMR without having been granted access	EAccessNotFound
T-SC-ILL04	Healthcare personnel attempts to read both the administrative and clinical sections of a patient’s EMR after the allowed time has expired	EAccessExpired
T-SC-ILL05	Healthcare personnel attempts to read both the administrative and clinical sections of a patient’s EMR without having been granted access	EAccessNotFound
T-SC-ILL06	Healthcare personnel attempts to perform a write operation by adding a new EMR entry after the allowed time has expired	EAccessExpired
T-SC-ILL07	Healthcare personnel attempts to perform a write operation by adding a new EMR entry after already adding one within the same access period	EMedicalRecordCreationLimit
T-SC-ILL08	Healthcare personnel attempts to perform a write operation by adding a new EMR entry for a patient without having been granted access	EAccessNotFound
T-SC-ILL09	Healthcare personnel attempts to perform a write operation by editing an EMR entry after the allowed time has expired	EAccessExpired
T-SC-ILL10	Healthcare personnel attempts to perform a write operation by editing a patient’s EMR entry without having been granted access	EAccessNotFound
T-SC-ILL11	Healthcare personnel attempts to perform a write operation by editing an EMR entry created by another healthcare personnel	–

## Data Availability

The data presented in this study are available on request from the corresponding author.
